# The crystal structures of three pyrazine-2,5-dicarb­oxamides: three-dimensional supra­molecular structures

**DOI:** 10.1107/S2056989017005898

**Published:** 2017-04-21

**Authors:** Dilovan S. Cati, Helen Stoeckli-Evans

**Affiliations:** a Debiopharm International S.A., Chemin Messidor 5-7, CP 5911, CH-1002 Lausanne, Switzerland; b Institute of Physics, University of Neuchâtel, rue Emile-Argand 11, CH-2000 Neuchâtel, Switzerland

**Keywords:** crystal structure, dicarboxamide, pyrazine, pyridine, hydrogen bonding, offset π–π inter­actions, supra­molecular three-dimensional structure

## Abstract

The complete mol­ecules of the title compounds, *N* ^2^,*N* ^5^-bis­(pyridin-2-ylmeth­yl)pyrazine-2,5-dicarboxamide, C_18_H_16_N_6_O_2_ (I), 3,6-dimethyl-*N* ^2^,*N* ^5^-bis­(pyridin-2-yl­meth­yl)pyrazine-2,5-dicarboxamide, C_20_H_20_N_6_O_2_ (II), and *N* ^2^,*N* ^5^-bis­(pyridin-4-ylmeth­yl)pyrazine-2,5-dicarboxamide, C_18_H_16_N_6_O_2_ (III), are generated by inversion symmetry, with the pyrazine rings being located about centres of inversion. Each mol­ecule has an extended conformation with the pyridine rings inclined to the pyrazine ring by 89.17 (7)° in (I), 75.83 (8)° in (II) and by 82.71 (6)° in (III). In the crystal of (I), mol­ecules are linked by N—H⋯N hydrogen bonds, forming layers lying parallel to the *bc* plane. The layers are linked by C—H⋯O hydrogen bonds, forming a three-dimensional supra­molecular structure. In the crystal of (II), mol­ecules are also linked by N—H⋯N hydrogen bonds, forming layers lying parallel to the (10-1) plane. As in (I), the layers are linked by C—H⋯O hydrogen bonds, forming a three-dimensional supra­molecular structure. In the crystal of (III), mol­ecules are again linked by N—H⋯N hydrogen bonds, but here form corrugated sheets lying parallel to the *bc* plane. Within the sheets, neighbouring pyridine rings are linked by offset π–π inter­actions [inter­centroid distance = 3.739 (1) Å]. The sheets are linked by C—H⋯O hydrogen bonds, forming a three-dimensional supra­molecular structure. Compound (I) crystallizes in the monoclinic space group *P*2_1_/*c*. Another monoclinic polymorph, space group *C*2/*c*, has been reported on by Cockriel *et al.* [*Inorg. Chem. Commun.* (2008), **11**, 1–4]. The mol­ecular structures of the two polymorphs are compared.

## Abstract 2

The whole mol­ecules of the three title pyrazine-2,5-dicarboxamide compounds are generated by inversion symmetry. Each mol­ecule has an extended conformation with the pyridine rings being inclined to the pyrazine ring by 89.17 (7), 75.83 (8) and 82.71 (6)°.

## Chemical context   

The title compounds are part of a series of pyrazine mono- and di- and tetra­kis­carboxamide derivatives synthesized to study their coordination chemistry with essentially first-row trans­ition metals (Cati, 2002 ▸). Compound (I) crystallizes in the monoclinic space group *P*2_1_/*c*. Another monoclinic polymorph, space group *C*2/*c*, has been described by Cockriel *et al.* (2008 ▸).

## Structural commentary   

**Figure 1:**
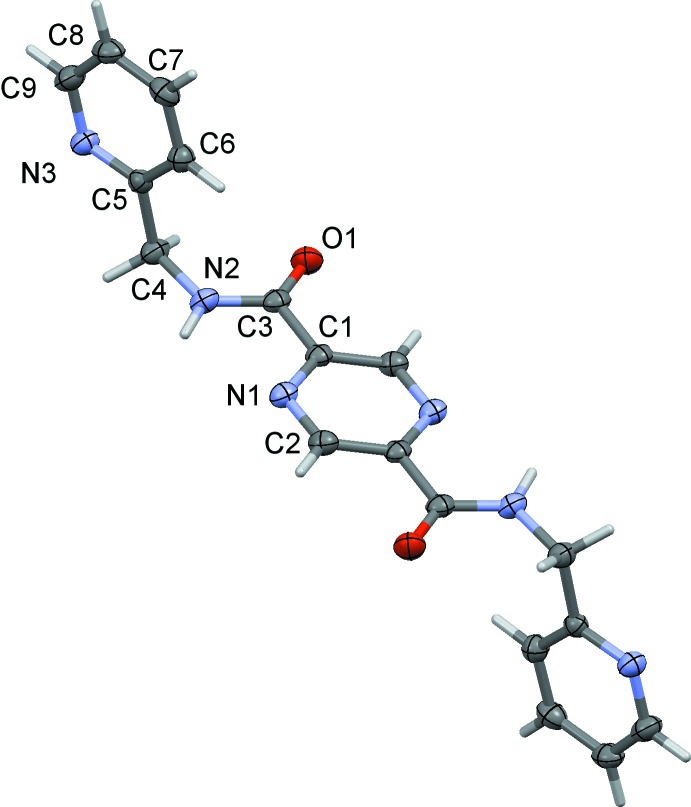
A view of the mol­ecular structure of compound (I), with the atom labelling. Displacement ellipsoids are drawn at the 50% probability level. The unlabelled atoms are related to the labelled atoms by inversion symmetry (symmetry operation: −*x*, −*y*, −*z* + 2).

The mol­ecular structures of the title compounds, (I), (II) and (III), are illustrated in Figs. 1 ▸, 2 ▸ and 3 ▸, respectively. The whole mol­ecule of each compound is generated by inversion symmetry, with the pyrazine rings being located about centers of inversion. Each mol­ecule has an extended conformation with the pyridine rings inclined to the pyrazine ring by 89.17 (7)° in (I), by 75.83 (8)° in (II) and by 82.71 (6)° in (III). The methyl­carboxamide units (C4—N2—C3=O1) are inclined to the pyrazine ring by 4.24 (9), 3.13 (10) and 9.32 (8)° in (I), (II) and (III), respectively.
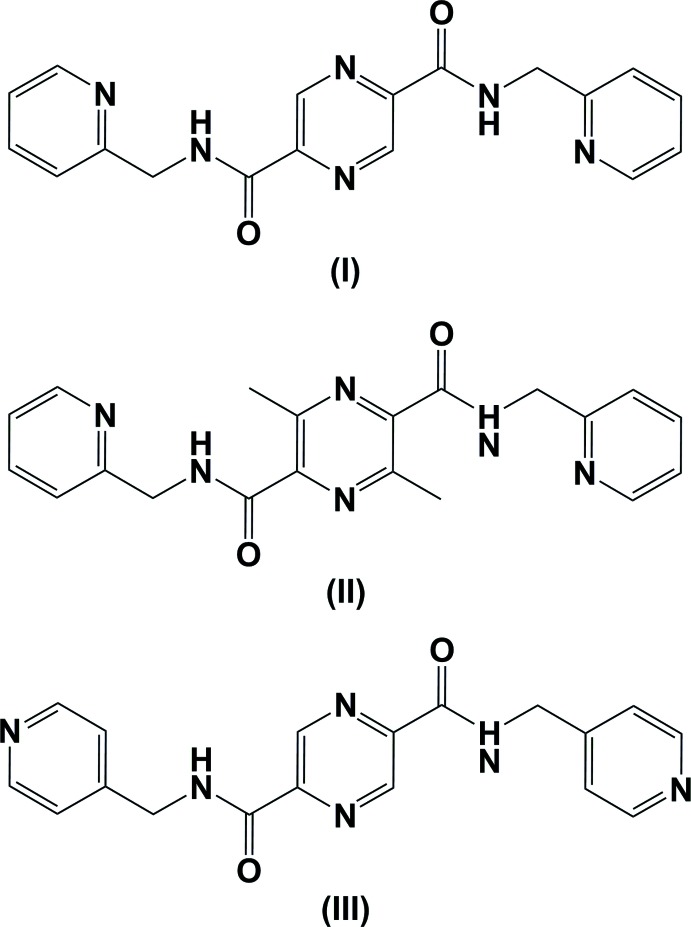


**Figure 4:**
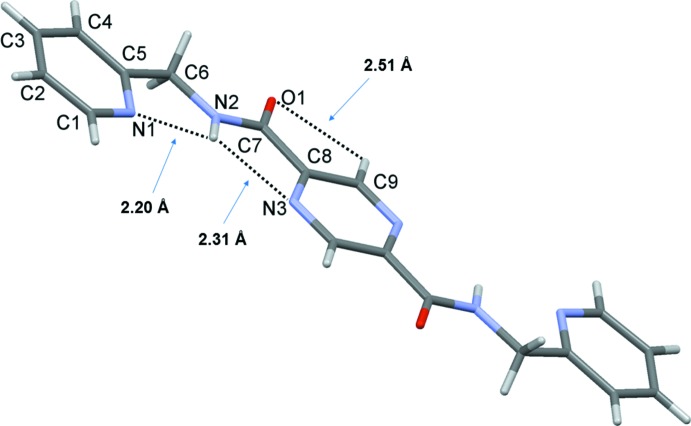
A view of the mol­ecular structure of the monoclinic *C*2/*c* polymorph (Cockriel *et al.*, 2008 ▸) of compound (I), with the atom labelling. The unlabelled atoms are related to the labelled atoms by inversion symmetry (symmetry operation: −*x* + 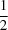, −*y* − 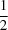, −*z* + 1).

In the monoclinic *C*2/*c* polymorph of (I) (Cockriel *et al.*, 2008 ▸), the whole mol­ecule is also generated by inversion symmetry (Fig. 4 ▸). However, here the mol­ecule is almost planar with the pyridine rings being inclined to the pyrazine ring by only 5.70 (7)°. The pyridine ring is orientated in such a manner that the NH hydrogen atom forms short contacts with both the adjacent pyrazine and pyridine N atoms, as shown in Fig. 4 ▸. The carbonyl O atom also accepts a short contact from a pyrazine H atom (Fig. 4 ▸).

## Supra­molecular features   

**Figure 5:**
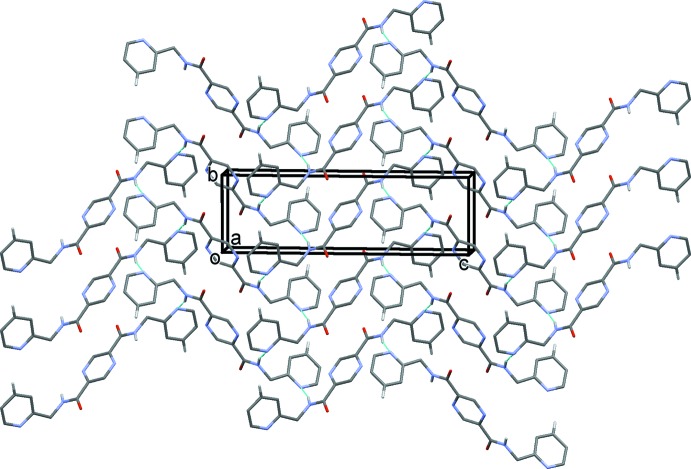
A view along the *a* axis of the crystal pack of compound (I). The N—H⋯N hydrogen bonds are shown as dashed lines (see Table 1 ▸). For clarity, in this and subsequent figures, only the H atoms involved in hydrogen bonding have been included.

**Figure 6:**
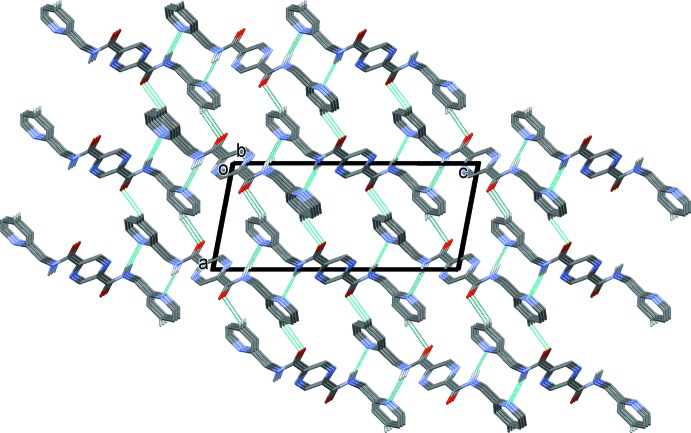
A view along the *b* axis of the crystal pack of compound (I). The hydrogen bonds are shown as dashed lines (see Table 1 ▸).

In the crystal of (I), mol­ecules are linked by N—H⋯N hydrogen bonds, forming layers lying parallel to the *bc* plane (Table 1 ▸ and Fig. 5 ▸). The layers are linked by C—H⋯O hydrogen bonds, forming a three-dimensional supra­molecular structure (Table 1 ▸ and Fig. 6 ▸)

**Figure 7:**
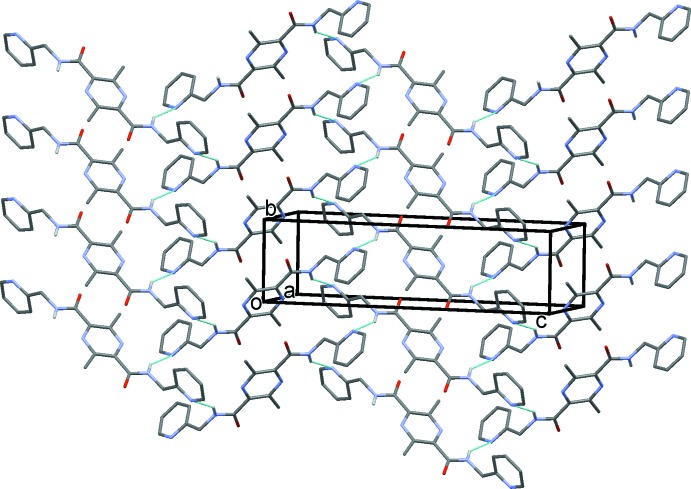
A view along the normal to plane (10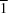), of the crystal pack of compound (II). The N—H⋯N hydrogen bonds are shown as dashed lines (see Table 2 ▸).

**Figure 8:**
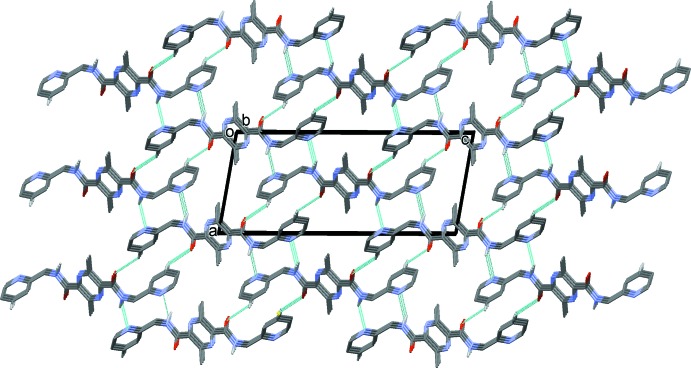
A view along the *b* axis of the crystal pack of compound (II). The hydrogen bonds are shown as dashed lines (see Table 2 ▸).

In the crystal of (II), mol­ecules are linked by N—H⋯N hydrogen bonds, forming layers lying parallel to the (10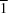) plane (Table 2 ▸ and Fig. 7 ▸). As in the crystal of (I), the layers are linked by C—H⋯O hydrogen bonds, forming a three-dimensional supra­molecular structure (Table 2 ▸ and Fig. 8 ▸)

**Figure 9:**
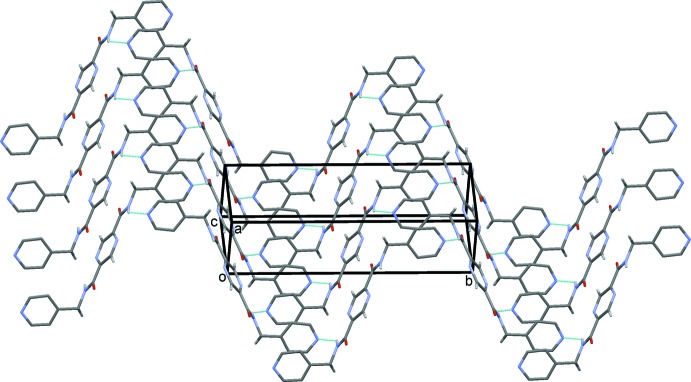
A partial view, normal to plane (10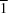), of the crystal pack of compound (III). The N—H⋯N hydrogen bonds are shown as dashed lines (see Table 3 ▸).

**Figure 10:**
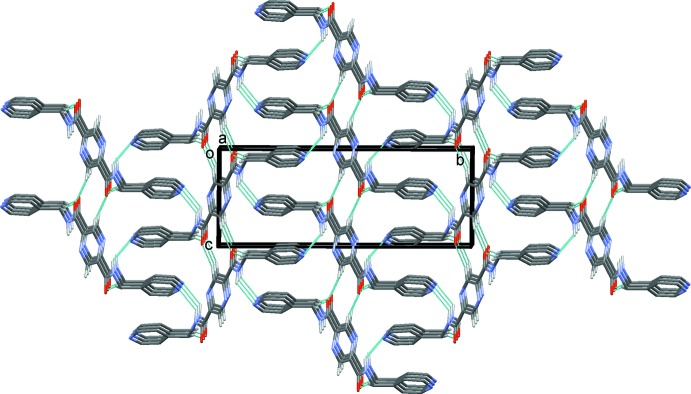
A view along the *a* axis of the crystal pack of compound (III). The hydrogen bonds are shown as dashed lines (see Table 3 ▸).

In the crystal of (III), mol­ecules are linked by N—H⋯N hydrogen bonds, forming corrugated sheets lying parallel to the *bc* plane (Table 3 ▸ and Fig. 9 ▸). The sheets are linked by C—H⋯O hydrogen bonds, forming a three-dimensional supra­molecular structure (Table 3 ▸ and Fig. 10 ▸). Within the sheets, neighbouring pyridine rings are linked by offset π–π inter­actions [*Cg*2⋯*Cg*2^iv^ = 3.739 (1) Å, *Cg*2 is the centroid of the pyridine ring (N3/C5–C9), α = 1.85 (7)°, inter­planar distances = 3.525 (1) and 3.552 (1) Å, slippage = 1.168 Å; symmetry code: (iv) *x* + 1, −*y* + 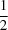, *z* + 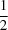].

## Database survey   

A search of the Cambridge Structural Database (Version 5.38, update February 2017; Groom *et al.*, 2016 ▸) for pyrazine-2,5-dicarboxamides yielded three hits, *viz. N,N′*-bis­(4-pentyl­phen­yl)pyrazine-2,5-dicarboxamide (CSD refcode: DABDOC; Zhang *et al.*, 2015 ▸), *N,N′*-di­phenyl­pyrazine-2,5-dicarboxamide (HIYKEH; Cheng *et al.*, 2014 ▸), and the monoclinic *C*2/*c* polymorph of compound (I) (AFAPOV; Cockriel *et al.*, 2008 ▸), mentioned above. All three compounds possess inversion symmetry and HIYKEH, like AFAPOV, has an almost planar conformation (*cf*. Fig. 4 ▸).

## Synthesis and crystallization   

**Pyrazine 2,5-di­carb­oxy­lic acid** was prepared by oxidation of 2,5-di­methyl­pyrazine with selenium dioxide (Schut *et al.*, 1961 ▸).

**Dimethyl 3,6-di­methyl­pyrazine-2,5-di­carboxyl­ate** was prepared following reported procedures (Takeuchi *et al.*, 1990 ▸; Wang, 1996 ▸).

**Dimethyl pyrazine-2,5-di­carboxyl­ate** was obtained following the procedure described by (Schut *et al.*, 1961 ▸). A mixture of anhydrous pyrazine-2,5-di­carb­oxy­lic acid (5 g, 30 mmol), absolute methanol (190 ml) and 1.5 ml (*ca* 3 g) of conc. sulfuric acid were refluxed until a clear solution was obtained (*ca* 9 h). After standing overnight at 268 K, the crystalline product formed was filtered off, washed with ice-cold methanol (2 × 20 ml) then dried over PO_5_ [yield 90%, m.p. 441 (1) K].

***Note*** **:** Both pyrazine 2,5-di­carb­oxy­lic acid and dimethyl pyrazine-2,5-di­carboxyl­ate are also available commercially.

**Compound (I):** was prepared by refluxing dimethyl pyrazine-2,5-di­carboxyl­ate (1.00 g, 5 mmol) and an excess of 2-(amino­meth­yl)pyridine (1.55g, 14.3 mmol) in 30 ml of methanol in a two-necked flask (100 ml). After 150 min a precipitate appeared, and after refluxing for 5 h the suspension was cooled to room temperature. A white solid was filtered off and washed with 10 ml of cold methanol. It was then recrystallized from di­chloro­methane solution to give colourless plate-like crystals of (I) suitable for X-ray diffraction analysis (yield 81%, m.p. 479 K).

Spectroscopic and analytical data:

^1^H NMR (400 MHz, DMSO-*d*_6_): 9.51 (*t*, 1H, *J*_hg_ = 5.9, Hh); 9.28 (*s*, 1H, Hl = Hn); 8.54 (*ddd*, 1H, *J*_bc_ = 4.8, *J*_bd_ = 1.8, *J*_be_ = 0.8, Hb); 7.76 (*td*, 1H, *J*_dc_ = 7.7, *J*_db_ = 1.8, Hd); 7.38 (*d*, 1H, *J*_ed_ = 7.8, He); 7.28 (*m*, 1H, Hc); 4.68 (*d*, 2H, *J*_gh_ = 5.9, Hg).

^13^C NMR (400 MHz, DMSO-*d*6): 163.4, 158.5, 149.7, 147.4, 143.0, 137.6, 123.1, 122.0, 45.2.

IR (KBr pellet, cm^−1^): 3335 (*s*), 3055 (*m*), 2916 (*w*), 1683 (*vs*), 1603 (*s*), 1593 (*s*), 1572 (*s*), 1522 (*vs*), 1483 (*s*), 1464 (*vs*), 1436 (*vs*), 1364 (*m*), 1328 (*s*), 1296 (*m*), 1255 (*m*), 1242 (*m*), 1210 (*m*), 1182 (*m*), 1148 (*m*), 1048 (*m*), 1028 (*m*), 1024 (*m*), 999 (*m*), 943 (*w*), 903 (*s*), 759 (*vs*), 728 (*m*), 668 (*m*), 506 (*s*), 461 (*s*).

Analysis for C_18_H_16_N_6_O_2_ (*M*_r_ = 348.36 g mol^−1^). Calculated (%) C: 62.06, H: 4.63, N: 24.12. Found (%) C: 62.00, H: 4.67, N: 24.30.

**Compound (II):** was prepared by refluxing dimethyl 3,6-di­methyl­pyrazine-2,5-di­carboxyl­ate (1.5 g, 5.92 mmol) and an excess of 2-(amino­meth­yl)pyridine (1.63 g, 15 mmol) in 25 ml of methanol, in a two-necked flask (100 ml) for 55 h. A colourless precipitate formed and this suspension was then cooled to room temperature. The solid that had formed was filtered off and washed with 10 ml of cold methanol. It was then recrystallized from ethyl acetate solution to give colourless rod-like crystals of (II) [yield 90%, m.p. 470 K].

Spectroscopic and analytical data:

^1^H NMR (400 MHz, DMSO-*d*_6_): 9.39 (*t*, 1H, *J*_hg_ = 6.1, Hh); 8.54 (*ddd*, 1H, *J*_bc_ = 4.8, *J*_bd_ = 1.8, *J*_be_ = 0.9, Hb); 7.79 (*td*, 1H, *J*_dc_ = 7.7, *J*_db_ = 1.8, Hd); 7.37 (*d*, 1H, *J*_ed_ = 7.9, He); 7.29 (*m*, 1H, Hc); 4.61 (*d*, 2H, *J*_gh_ = 6.1, Hg); 2.79 (*s*, 3H, CH_3_).

^13^C NMR (400 MHz, DMSO-*d*_6_): 165.5, 158.9, 149.8, 149.7, 145.3, 137.7, 123.1, 121.9, 45.2, 22.8.

IR (KBr pellet, cm^−1^): 3310 (*s*), 3090 (*m*), 3055 (*m*), 3011 (*m*), 2904 (*m*), 1673 (*vs*), 1609 (*m*), 1592 (*vs*), 1569 (*s*), 1506 (*vs*), 1474 (*vs*), 1435 (*vs*), 1411 (*vs*), 1372 (*m*), 1352 (*s*), 1275 (*s*), 1243 (*s*), 1185 (*s*), 1158 (*s*), 1092 (*m*), 1050 (*m*), 1033 (*w*), 1015 (*s*), 995 (*s*), 971 (*m*), 959 (*w*), 888 (*w*), 833 (*m*), 770 (*m*), 759 (*s*), 715 (*s*), 640 (*m*), 556 (*m*), 526 (*s*), 463 (*m*), 447 (*m*).

Analysis for C_20_H_20_N_6_O_2_ (*M*_r_ = 376.42 g mol^−1^). Calculated (%) C: 63.82, H: 5.36, N: 22.33. Found (%) C: 63.74, H: 5.46, N: 22.42.

**Compound (III):** was prepared by heating to reflux a mixture of dimethyl pyrazine-2,5-di­carboxyl­ate (1.00 g, 5 mmol) with an excess of 4-(amino­meth­yl)pyridine (1.55g, 14.3 mmol) in 35 ml of methanol in a two-necked flask (100 ml). After 6 h the white solid that had formed was filtered off and washed with 10 ml of cold methanol. It was then recrystallized from di­chloro­methane solution to give colourless block-like crystals of (III) [yield 85%, m.p. 530 K (degradation)].

Spectroscopic and analytical data:

^1^H NMR (400 MHz, DMSO-*d*_6_): 9.80 (*t*, 1H, *J*_hg_ = 6.3, Hh); 9.25 (*s*, 1H, Hn = Hl); 8.50 (*dd*, 2H, *J*_ba_ = 4.5, *J*_be_ = 1.5, Hb = Hd); 7.33 (*dd*, 2H, *J*_ab_ = 4.5, *J*_eb_ = 1.5, Ha = He); 4.55 (*d*, 2H, *J*_gh_ = 6.3, Hg).

^13^C NMR (400 MHz, DMSO-*d*_6_): 163.7, 150.4, 148.8, 147.4, 143.0, 123.1, 42.5.

IR (KBr pellet, cm^−1^): 3348 (*s*), 3089 (*w*), 3073 (*w*), 3032 (*w*), 2997 (*w*), 2934 (*w*), 2359 (*w*), 1949 (*w*), 1712 (*m*), 1662 (*vs*), 1604 (*s*), 1561 (*s*), 1533 (*vs*), 1496 (*w*), 1472 (*m*), 1427 (*s*), 1418 (*vs*), 1373 (*m*), 1317 (*w*), 1282 (*s*), 1233 (*w*), 1220 (*w*), 1204 (*w*), 1171 (*s*), 1135 (*w*), 1067 (*w*), 1028 (*m*), 991 (*s*), 970 (*w*), 824 (*m*), 777 (*m*), 726 (*w*), 671 (*m*), 662 (*m*), 607 (*w*), 511 (*m*), 457 (*m*).

Analysis for C_18_H_16_N_6_O_2_·0.5CH_3_OH (*M2* = 364.39 g mol^−1^). Calculated (%) C: 60.98, H: 4.98, N: 23.06. Found (%) C: 61.12, H: 4.83, N: 22.85.

## Refinement   

Crystal data, data collection and structure refinement details are summarized in Table 4 ▸. Intensity data for (I) and (III) were measured at 153 K on a one-circle image-plate diffractometer, while for (II) intensity data were measured at 293 K on a four-circle diffractometer. For all three compounds, the NH H atoms were located in difference-Fourier maps and freely refined. The C-bound H atoms were included in calculated positions and treated as riding: C—H = 0.95–0.99 Å for (I) and (III) with *U*_iso_(H) = 1.2*U*_eq_(C); C—H = 0.93–0.96 Å for (II), with *U*_iso_(H) = 1.5*U*_eq_(C-meth­yl) and 1.2*U*_eq_(C) for other H atoms.

## Supplementary Material

Crystal structure: contains datablock(s) I, II, III, Global. DOI: 10.1107/S2056989017005898/hb7672sup1.cif

Structure factors: contains datablock(s) I. DOI: 10.1107/S2056989017005898/hb7672Isup2.hkl

Structure factors: contains datablock(s) II. DOI: 10.1107/S2056989017005898/hb7672IIsup3.hkl

Structure factors: contains datablock(s) III. DOI: 10.1107/S2056989017005898/hb7672IIIsup4.hkl

Supporting information file. DOI: 10.1107/S2056989017005898/hb7672Isup5.cml

Supporting information file. DOI: 10.1107/S2056989017005898/hb7672IIsup6.cml

Supporting information file. DOI: 10.1107/S2056989017005898/hb7672IIIsup7.cml

CCDC references: 1544871, 1544870, 1544869

Additional supporting information:  crystallographic information; 3D view; checkCIF report

## Figures and Tables

**Figure 2:**
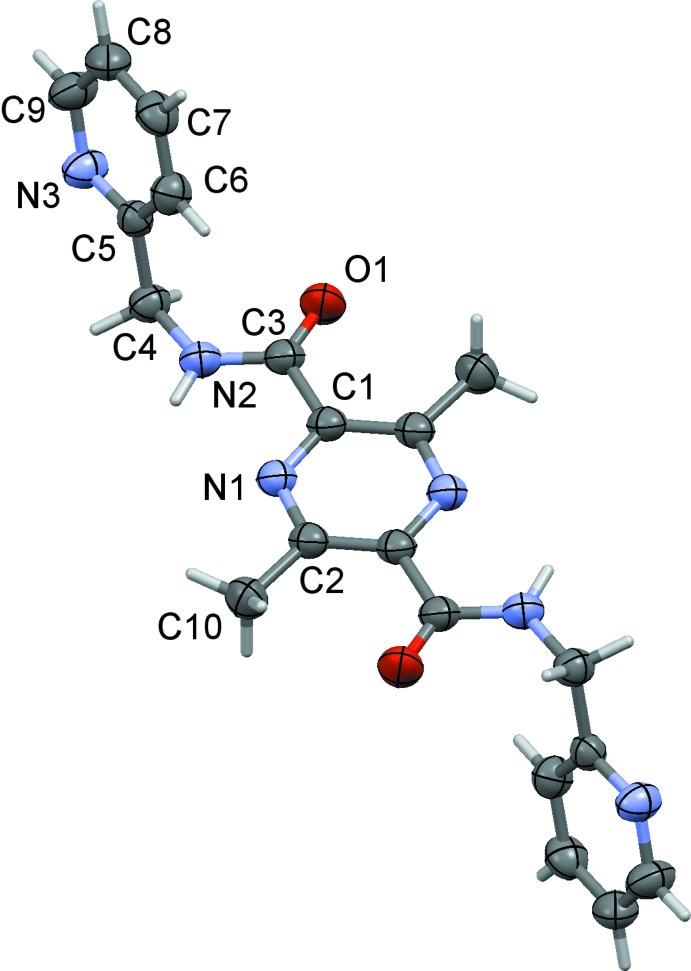
A view of the mol­ecular structure of compound (II), with the atom labelling. Displacement ellipsoids are drawn at the 50% probability level. The unlabelled atoms are related to the labelled atoms by inversion symmetry (symmetry operation: −*x*, −*y*, −*z*).

**Figure 3:**
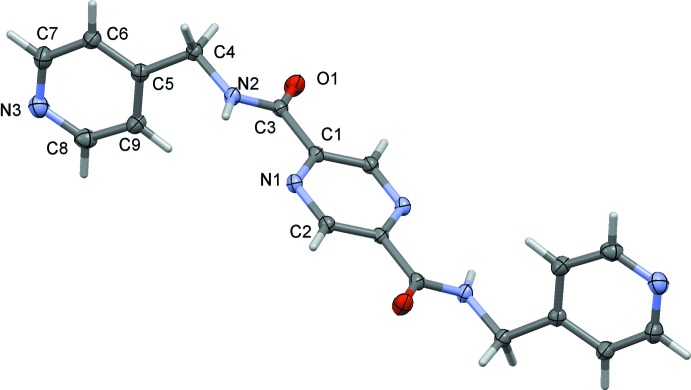
A view of the mol­ecular structure of compound (III), with the atom labelling. Displacement ellipsoids are drawn at the 50% probability level. The unlabelled atoms are related to the labelled atoms by inversion symmetry (symmetry operation: −*x* + 1, −*y* + 1, −*z* + 2).

**Table 1:** Hydrogen-bond geometry (Å, °) for (I)

*D*—H⋯*A*	*D*—H	H⋯*A*	*D*⋯*A*	*D*—H⋯*A*
N2—H2*N*⋯N3^i^	0.88 (2)	2.209 (17)	3.0657 (18)	164 (2)
C7—H7⋯O1^ii^	0.95	2.53	3.292 (2)	137

Symmetry codes: (i) 
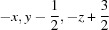
; (ii) 
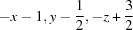
.

**Table 2:** Hydrogen-bond geometry (Å, °) for (II)

*D*—H⋯*A*	*D*—H	H⋯*A*	*D*⋯*A*	*D*—H⋯*A*
N2—H2*N*⋯N3^i^	0.85 (2)	2.35 (2)	3.097 (2)	147.4 (18)
C7—H7⋯O1^ii^	0.93	2.59	3.263 (2)	130

Symmetry codes: (i) 
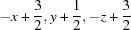
; (ii) 
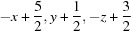
.

**Table 3:** Hydrogen-bond geometry (Å, °) for (III)

*D*—H⋯*A*	*D*—H	H⋯*A*	*D*⋯*A*	*D*—H⋯*A*
N2—H2*N*⋯N3^i^	0.93 (2)	2.50 (2)	3.2420 (19)	137.5 (16)
C2—H2⋯O1^ii^	0.95	2.33	3.2411 (18)	160
C4—H4*B*⋯O1^iii^	0.99	2.49	3.4636 (18)	166

Symmetry codes: (i) 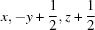; (ii) 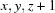; (iii) 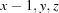.

**Table 4:** Experimental details

	(I)	(II)	(III)
Crystal data			
Chemical formula	C_18_H_16_N_6_O_2_	C_20_H_20_N_6_O_2_	C_18_H_16_N_6_O_2_
*M*_r_	348.37	376.42	348.37
Crystal system, space group	Monoclinic, *P*2_1_/*c*	Monoclinic, *P*2_1_/*n*	Monoclinic, *P*2_1_/*c*
Temperature (K)	153	293	153
*a*, *b*, *c* (Å)	8.0769 (9), 5.6076 (7), 18.3724 (18)	8.7271 (5), 5.2950 (4), 20.1403 (13)	5.8663 (6), 18.7539 (17), 7.2943 (8)
β (°)	100.781 (12)	99.834 (6)	101.606 (12)
*V* (Å^3^)	817.44 (16)	917.01 (11)	786.08 (14)
*Z*	2	2	2
Radiation type	Mo *K*α	Cu *K*α	Mo *K*α
μ (mm^−1^)	0.10	0.75	0.10
Crystal size (mm)	0.45 × 0.25 × 0.15	0.46 × 0.19 × 0.19	0.35 × 0.30 × 0.25

Data collection			
Diffractometer	Stoe *IPDS* 1	Stoe–Siemens AED2 four-circle	Stoe *IPDS* 1
Absorption correction	Multi-scan (*MULABS*; Spek, 2009 ▸)	Multi-scan (*MULABS*; Spek, 2009 ▸)	Multi-scan (*MULABS*; Spek, 2009 ▸)
*T*_min_, *T*_max_	0.987, 1.000	0.955, 1.000	0.962, 1.000
No. of measured, independent and observed [*I* > 2σ(*I*)] reflections	5998, 1574, 1024	2576, 1345, 1226	5980, 1513, 1259
*R*_int_	0.037	0.015	0.026
θ_max_ (°)	25.9	59.6	25.9
(sin θ/λ)_max_ (Å^−1^)	0.615	0.559	0.615

Refinement			
*R*[*F* ^2^ > 2σ(*F* ^2^)], *wR*(*F* ^2^), *S*	0.032, 0.076, 0.87	0.035, 0.095, 1.05	0.036, 0.097, 1.08
No. of reflections	1574	1345	1513
No. of parameters	123	133	122
H-atom treatment	H atoms treated by a mixture of independent and constrained refinement	H atoms treated by a mixture of independent and constrained refinement	H atoms treated by a mixture of independent and constrained refinement
Δρ_max_, Δρ_min_ (e Å^−3^)	0.16, −0.15	0.18, −0.12	0.44, −0.19

Computer programs: *EXPOSE*, *CELL* and *INTEGRATE* in *IPDS-I* (Stoe & Cie, 2004 ▸), *STADI4* (Stoe & Cie, 1997 ▸), *X-RED* (Stoe & Cie, 1997 ▸), *SHELXS97* (Sheldrick, 2008 ▸), *SHELXL2014* and *SHELXL2016* (Sheldrick, 2015 ▸), *PLATON* (Spek, 2009 ▸), *Mercury* (Macrae *et al.*, 2008 ▸) and *publCIF* (Westrip, 2010 ▸).

## supplementary crystallographic information

### (I) N2,N5-Bis(pyridin-2-ylmethyl)pyrazine-2,5-dicarboxamide Crystal data

**Table:**

C_18_H_16_N_6_O_2_	*F*(000) = 364
*M**_r_* = 348.37	*D*_x_ = 1.415 Mg m^−^^3^
Monoclinic, *P*2_1_/*c*	Mo *K*α radiation, λ = 0.71073 Å
*a* = 8.0769 (9) Å	Cell parameters from 3344 reflections
*b* = 5.6076 (7) Å	θ = 2.3–25.9°
*c* = 18.3724 (18) Å	µ = 0.10 mm^−^^1^
β = 100.781 (12)°	*T* = 153 K
*V* = 817.44 (16) Å^3^	Plate, colourless
*Z* = 2	0.45 × 0.25 × 0.15 mm

### (I) N2,N5-Bis(pyridin-2-ylmethyl)pyrazine-2,5-dicarboxamide Data collection

**Table:**

Stoe IPDS 1 diffractometer	1574 independent reflections
Radiation source: fine-focus sealed tube	1024 reflections with *I* > 2σ(*I*)
Plane graphite monochromator	*R*_int_ = 0.037
φ rotation scans	θ_max_ = 25.9°, θ_min_ = 2.3°
Absorption correction: multi-scan (MULABS; Spek, 2009)	*h* = −9→9
*T*_min_ = 0.987, *T*_max_ = 1.000	*k* = −6→6
5998 measured reflections	*l* = −22→22

### (I) N2,N5-Bis(pyridin-2-ylmethyl)pyrazine-2,5-dicarboxamide Refinement

**Table:**

Refinement on *F*^2^	Secondary atom site location: difference Fourier map
Least-squares matrix: full	Hydrogen site location: mixed
*R*[*F*^2^ > 2σ(*F*^2^)] = 0.032	H atoms treated by a mixture of independent and constrained refinement
*wR*(*F*^2^) = 0.076	*w* = 1/[σ^2^(*F*_o_^2^) + (0.0456*P*)^2^] where *P* = (*F*_o_^2^ + 2*F*_c_^2^)/3
*S* = 0.87	(Δ/σ)_max_ < 0.001
1574 reflections	Δρ_max_ = 0.16 e Å^−^^3^
123 parameters	Δρ_min_ = −0.15 e Å^−^^3^
0 restraints	Extinction correction: SHELXL2016 (Sheldrick, 2015), Fc^*^=kFc[1+0.001xFc^2^λ^3^/sin(2θ)]^-1/4^
Primary atom site location: structure-invariant direct methods	Extinction coefficient: 0.029 (3)

### (I) N2,N5-Bis(pyridin-2-ylmethyl)pyrazine-2,5-dicarboxamide Special details

**Table:**

Geometry. All esds (except the esd in the dihedral angle between two l.s. planes) are estimated using the full covariance matrix. The cell esds are taken into account individually in the estimation of esds in distances, angles and torsion angles; correlations between esds in cell parameters are only used when they are defined by crystal symmetry. An approximate (isotropic) treatment of cell esds is used for estimating esds involving l.s. planes.

### (I) N2,N5-Bis(pyridin-2-ylmethyl)pyrazine-2,5-dicarboxamide Fractional atomic coordinates and isotropic or equivalent isotropic displacement parameters (Å^2^)

**Table:**

	*x*	*y*	*z*	*U*_iso_*/*U*_eq_	
N1	0.05799 (15)	0.0574 (2)	0.93513 (7)	0.0261 (3)	
N2	−0.05730 (16)	0.4484 (2)	0.85602 (7)	0.0274 (3)	
H2N	0.031 (2)	0.366 (3)	0.8485 (10)	0.045 (5)*	
N3	−0.26574 (14)	0.7526 (2)	0.68676 (7)	0.0274 (3)	
O1	−0.24558 (13)	0.51737 (18)	0.93146 (6)	0.0336 (3)	
C1	−0.06037 (17)	0.1872 (2)	0.95909 (8)	0.0232 (3)	
C2	0.11771 (18)	−0.1299 (3)	0.97651 (8)	0.0262 (4)	
H2A	0.201781	−0.226769	0.961478	0.031*	
C3	−0.13034 (17)	0.4004 (3)	0.91401 (8)	0.0253 (4)	
C4	−0.10976 (18)	0.6452 (3)	0.80607 (8)	0.0283 (4)	
H4A	−0.009417	0.709655	0.789038	0.034*	
H4B	−0.155051	0.773295	0.833863	0.034*	
C5	−0.24122 (16)	0.5825 (3)	0.73894 (8)	0.0223 (3)	
C6	−0.33024 (18)	0.3704 (3)	0.73168 (9)	0.0278 (4)	
H6	−0.308875	0.252378	0.769361	0.033*	
C7	−0.45098 (18)	0.3331 (3)	0.66858 (9)	0.0317 (4)	
H7	−0.513660	0.188834	0.662344	0.038*	
C8	−0.47904 (19)	0.5068 (3)	0.61521 (9)	0.0323 (4)	
H8	−0.561978	0.485946	0.571644	0.039*	
C9	−0.38401 (19)	0.7125 (3)	0.62623 (9)	0.0332 (4)	
H9	−0.403298	0.832226	0.589013	0.040*	

### (I) N2,N5-Bis(pyridin-2-ylmethyl)pyrazine-2,5-dicarboxamide Atomic displacement parameters (Å^2^)

**Table:**

	*U*^11^	*U*^22^	*U*^33^	*U*^12^	*U*^13^	*U*^23^
N1	0.0263 (6)	0.0271 (7)	0.0233 (7)	0.0005 (5)	0.0001 (5)	0.0007 (6)
N2	0.0282 (7)	0.0282 (7)	0.0240 (7)	0.0038 (6)	0.0001 (6)	0.0040 (6)
N3	0.0286 (7)	0.0269 (7)	0.0256 (7)	0.0020 (6)	0.0024 (6)	0.0033 (6)
O1	0.0294 (6)	0.0338 (6)	0.0371 (7)	0.0068 (5)	0.0051 (5)	0.0049 (5)
C1	0.0216 (7)	0.0249 (8)	0.0210 (8)	−0.0027 (6)	−0.0018 (6)	−0.0015 (6)
C2	0.0244 (7)	0.0269 (8)	0.0257 (8)	0.0023 (6)	0.0006 (6)	−0.0024 (7)
C3	0.0229 (7)	0.0262 (8)	0.0240 (8)	−0.0019 (6)	−0.0026 (6)	−0.0006 (7)
C4	0.0298 (8)	0.0260 (8)	0.0265 (9)	−0.0019 (6)	−0.0012 (7)	0.0036 (7)
C5	0.0207 (7)	0.0243 (8)	0.0222 (8)	0.0032 (6)	0.0046 (6)	0.0001 (6)
C6	0.0283 (8)	0.0270 (8)	0.0278 (9)	−0.0013 (6)	0.0048 (6)	0.0022 (7)
C7	0.0247 (8)	0.0341 (9)	0.0365 (10)	−0.0053 (7)	0.0058 (7)	−0.0071 (8)
C8	0.0245 (8)	0.0433 (10)	0.0267 (9)	0.0011 (7)	−0.0014 (6)	−0.0057 (8)
C9	0.0332 (9)	0.0380 (9)	0.0258 (9)	0.0062 (7)	−0.0014 (7)	0.0049 (7)

### (I) N2,N5-Bis(pyridin-2-ylmethyl)pyrazine-2,5-dicarboxamide Geometric parameters (Å, º)

**Table:**

N1—C2	1.3330 (19)	C4—C5	1.5107 (19)
N1—C1	1.3397 (18)	C4—H4A	0.9900
N2—C3	1.338 (2)	C4—H4B	0.9900
N2—C4	1.4475 (19)	C5—C6	1.383 (2)
N2—H2N	0.879 (19)	C6—C7	1.384 (2)
N3—C5	1.3405 (18)	C6—H6	0.9500
N3—C9	1.3424 (19)	C7—C8	1.370 (2)
O1—C3	1.2290 (17)	C7—H7	0.9500
C1—C2^i^	1.387 (2)	C8—C9	1.380 (2)
C1—C3	1.503 (2)	C8—H8	0.9500
C2—H2A	0.9500	C9—H9	0.9500
			
C2—N1—C1	116.42 (13)	C5—C4—H4B	108.6
C3—N2—C4	122.52 (13)	H4A—C4—H4B	107.6
C3—N2—H2N	120.6 (12)	N3—C5—C6	122.56 (13)
C4—N2—H2N	116.8 (12)	N3—C5—C4	113.93 (12)
C5—N3—C9	117.41 (13)	C6—C5—C4	123.51 (13)
N1—C1—C2^i^	121.83 (13)	C5—C6—C7	118.86 (14)
N1—C1—C3	117.97 (13)	C5—C6—H6	120.6
C2^i^—C1—C3	120.21 (13)	C7—C6—H6	120.6
N1—C2—C1^i^	121.75 (13)	C8—C7—C6	119.23 (14)
N1—C2—H2A	119.1	C8—C7—H7	120.4
C1^i^—C2—H2A	119.1	C6—C7—H7	120.4
O1—C3—N2	124.66 (14)	C7—C8—C9	118.44 (14)
O1—C3—C1	120.38 (14)	C7—C8—H8	120.8
N2—C3—C1	114.96 (13)	C9—C8—H8	120.8
N2—C4—C5	114.69 (12)	N3—C9—C8	123.49 (15)
N2—C4—H4A	108.6	N3—C9—H9	118.3
C5—C4—H4A	108.6	C8—C9—H9	118.3
N2—C4—H4B	108.6		
			
C2—N1—C1—C2^i^	0.0 (2)	C9—N3—C5—C6	1.1 (2)
C2—N1—C1—C3	179.79 (12)	C9—N3—C5—C4	−178.26 (13)
C1—N1—C2—C1^i^	0.0 (2)	N2—C4—C5—N3	−168.30 (13)
C4—N2—C3—O1	−1.0 (2)	N2—C4—C5—C6	12.3 (2)
C4—N2—C3—C1	179.29 (12)	N3—C5—C6—C7	−0.9 (2)
N1—C1—C3—O1	175.99 (13)	C4—C5—C6—C7	178.46 (14)
C2^i^—C1—C3—O1	−4.2 (2)	C5—C6—C7—C8	0.0 (2)
N1—C1—C3—N2	−4.33 (18)	C6—C7—C8—C9	0.6 (2)
C2^i^—C1—C3—N2	175.43 (12)	C5—N3—C9—C8	−0.5 (2)
C3—N2—C4—C5	−92.36 (17)	C7—C8—C9—N3	−0.3 (2)

Symmetry code: (i) −*x*, −*y*, −*z*+2.

### (I) N2,N5-Bis(pyridin-2-ylmethyl)pyrazine-2,5-dicarboxamide Hydrogen-bond geometry (Å, º)

**Table:**

*D*—H···*A*	*D*—H	H···*A*	*D*···*A*	*D*—H···*A*
N2—H2*N*···N3^ii^	0.88 (2)	2.209 (17)	3.0657 (18)	164 (2)
C7—H7···O1^iii^	0.95	2.53	3.292 (2)	137

Symmetry codes: (ii) −*x*, *y*−1/2, −*z*+3/2; (iii) −*x*−1, *y*−1/2, −*z*+3/2.

### (II) 3,6-Dimethyl-N2,N5-bis(pyridin-2-ylmethyl)pyrazine-2,5-dicarboxamide Crystal data

**Table:**

C_20_H_20_N_6_O_2_	*F*(000) = 396
*M**_r_* = 376.42	*D*_x_ = 1.363 Mg m^−^^3^
Monoclinic, *P*2_1_/*n*	Cu *K*α radiation, λ = 1.54186 Å
*a* = 8.7271 (5) Å	Cell parameters from 22 reflections
*b* = 5.2950 (4) Å	θ = 20.4–32.0°
*c* = 20.1403 (13) Å	µ = 0.75 mm^−^^1^
β = 99.834 (6)°	*T* = 293 K
*V* = 917.01 (11) Å^3^	Rod, colourless
*Z* = 2	0.46 × 0.19 × 0.19 mm

### (II) 3,6-Dimethyl-N2,N5-bis(pyridin-2-ylmethyl)pyrazine-2,5-dicarboxamide Data collection

**Table:**

Stoe–Siemens AED2 four-circle diffractometer	1226 reflections with *I* > 2σ(*I*)
Radiation source: fine-focus sealed tube	*R*_int_ = 0.015
Plane graphite monochromator	θ_max_ = 59.6°, θ_min_ = 4.5°
ω/2θ scans	*h* = −9→9
Absorption correction: multi-scan (MULABS; Spek, 2009)	*k* = 0→5
*T*_min_ = 0.955, *T*_max_ = 1.000	*l* = −22→22
2576 measured reflections	2 standard reflections every 60 min
1345 independent reflections	intensity decay: 2%

### (II) 3,6-Dimethyl-N2,N5-bis(pyridin-2-ylmethyl)pyrazine-2,5-dicarboxamide Refinement

**Table:**

Refinement on *F*^2^	Secondary atom site location: difference Fourier map
Least-squares matrix: full	Hydrogen site location: mixed
*R*[*F*^2^ > 2σ(*F*^2^)] = 0.035	H atoms treated by a mixture of independent and constrained refinement
*wR*(*F*^2^) = 0.095	*w* = 1/[σ^2^(*F*_o_^2^) + (0.0453*P*)^2^ + 0.2943*P*] where *P* = (*F*_o_^2^ + 2*F*_c_^2^)/3
*S* = 1.05	(Δ/σ)_max_ < 0.001
1345 reflections	Δρ_max_ = 0.18 e Å^−^^3^
133 parameters	Δρ_min_ = −0.12 e Å^−^^3^
0 restraints	Extinction correction: SHELXL2016 (Sheldrick, 2015), Fc^*^=kFc[1+0.001xFc^2^λ^3^/sin(2θ)]^-1/4^
Primary atom site location: structure-invariant direct methods	Extinction coefficient: 0.0124 (10)

### (II) 3,6-Dimethyl-N2,N5-bis(pyridin-2-ylmethyl)pyrazine-2,5-dicarboxamide Special details

**Table:**

Geometry. All esds (except the esd in the dihedral angle between two l.s. planes) are estimated using the full covariance matrix. The cell esds are taken into account individually in the estimation of esds in distances, angles and torsion angles; correlations between esds in cell parameters are only used when they are defined by crystal symmetry. An approximate (isotropic) treatment of cell esds is used for estimating esds involving l.s. planes.

### (II) 3,6-Dimethyl-N2,N5-bis(pyridin-2-ylmethyl)pyrazine-2,5-dicarboxamide Fractional atomic coordinates and isotropic or equivalent isotropic displacement parameters (Å^2^)

**Table:**

	*x*	*y*	*z*	*U*_iso_*/*U*_eq_	
O1	1.12510 (14)	0.4700 (3)	0.90448 (6)	0.0615 (4)	
N1	0.88491 (14)	0.9651 (3)	0.94504 (6)	0.0422 (4)	
N2	0.89518 (17)	0.6199 (3)	0.85116 (7)	0.0466 (4)	
H2N	0.818 (2)	0.714 (4)	0.8529 (10)	0.061 (6)*	
N3	0.94177 (16)	0.3148 (3)	0.69204 (7)	0.0503 (4)	
C1	1.01225 (18)	0.8206 (3)	0.95440 (8)	0.0399 (4)	
C2	0.86917 (18)	1.1468 (3)	0.98964 (8)	0.0408 (4)	
C3	1.01729 (18)	0.6201 (3)	0.90156 (8)	0.0432 (4)	
C4	0.87726 (19)	0.4317 (3)	0.79852 (8)	0.0453 (4)	
H4A	0.767587	0.416493	0.779865	0.054*	
H4B	0.911490	0.270168	0.818461	0.054*	
C5	0.96522 (17)	0.4847 (3)	0.74167 (8)	0.0381 (4)	
C6	1.06331 (19)	0.6880 (3)	0.74049 (9)	0.0466 (5)	
H6	1.074867	0.807192	0.774909	0.056*	
C7	1.14401 (19)	0.7128 (4)	0.68778 (9)	0.0514 (5)	
H7	1.210827	0.848639	0.686234	0.062*	
C8	1.1248 (2)	0.5356 (4)	0.63781 (9)	0.0529 (5)	
H8	1.179711	0.545797	0.602164	0.064*	
C9	1.0223 (2)	0.3422 (4)	0.64172 (9)	0.0577 (5)	
H9	1.008001	0.223016	0.607303	0.069*	
C10	0.7223 (2)	1.3002 (4)	0.97432 (9)	0.0539 (5)	
H10A	0.672121	1.267830	0.928884	0.081*	
H10B	0.747288	1.476475	0.979389	0.081*	
H10C	0.653830	1.254180	1.004892	0.081*	

### (II) 3,6-Dimethyl-N2,N5-bis(pyridin-2-ylmethyl)pyrazine-2,5-dicarboxamide Atomic displacement parameters (Å^2^)

**Table:**

	*U*^11^	*U*^22^	*U*^33^	*U*^12^	*U*^13^	*U*^23^
O1	0.0552 (8)	0.0691 (9)	0.0594 (8)	0.0208 (7)	0.0074 (6)	−0.0159 (7)
N1	0.0423 (8)	0.0466 (8)	0.0392 (7)	0.0049 (6)	0.0106 (6)	−0.0015 (6)
N2	0.0454 (8)	0.0516 (9)	0.0439 (8)	0.0066 (7)	0.0105 (7)	−0.0085 (7)
N3	0.0541 (9)	0.0501 (9)	0.0494 (8)	−0.0114 (7)	0.0165 (7)	−0.0128 (7)
C1	0.0403 (9)	0.0436 (10)	0.0380 (8)	0.0016 (7)	0.0132 (7)	0.0003 (7)
C2	0.0405 (9)	0.0431 (9)	0.0406 (9)	0.0035 (7)	0.0125 (7)	0.0016 (7)
C3	0.0435 (9)	0.0483 (10)	0.0403 (9)	0.0035 (8)	0.0141 (7)	−0.0024 (8)
C4	0.0480 (9)	0.0456 (10)	0.0437 (9)	−0.0039 (8)	0.0119 (7)	−0.0069 (8)
C5	0.0342 (8)	0.0384 (9)	0.0410 (9)	0.0028 (7)	0.0047 (6)	−0.0026 (7)
C6	0.0479 (10)	0.0429 (10)	0.0486 (10)	−0.0050 (8)	0.0071 (8)	−0.0064 (8)
C7	0.0442 (10)	0.0527 (11)	0.0577 (11)	−0.0086 (8)	0.0101 (8)	0.0047 (9)
C8	0.0491 (10)	0.0644 (12)	0.0482 (10)	−0.0025 (9)	0.0167 (8)	0.0022 (9)
C9	0.0668 (12)	0.0616 (13)	0.0495 (10)	−0.0104 (10)	0.0232 (9)	−0.0164 (9)
C10	0.0476 (10)	0.0573 (12)	0.0553 (11)	0.0116 (9)	0.0047 (8)	−0.0047 (9)

### (II) 3,6-Dimethyl-N2,N5-bis(pyridin-2-ylmethyl)pyrazine-2,5-dicarboxamide Geometric parameters (Å, º)

**Table:**

O1—C3	1.2255 (19)	C4—H4B	0.9700
N1—C1	1.336 (2)	C5—C6	1.378 (2)
N1—C2	1.339 (2)	C6—C7	1.377 (2)
N2—C3	1.340 (2)	C6—H6	0.9300
N2—C4	1.444 (2)	C7—C8	1.365 (3)
N2—H2N	0.85 (2)	C7—H7	0.9300
N3—C5	1.335 (2)	C8—C9	1.371 (3)
N3—C9	1.336 (2)	C8—H8	0.9300
C1—C2^i^	1.404 (2)	C9—H9	0.9300
C1—C3	1.509 (2)	C10—H10A	0.9600
C2—C10	1.504 (2)	C10—H10B	0.9600
C4—C5	1.510 (2)	C10—H10C	0.9600
C4—H4A	0.9700		
			
C1—N1—C2	119.67 (13)	N3—C5—C4	114.19 (14)
C3—N2—C4	121.93 (15)	C6—C5—C4	123.79 (14)
C3—N2—H2N	120.4 (14)	C7—C6—C5	119.25 (16)
C4—N2—H2N	116.7 (14)	C7—C6—H6	120.4
C5—N3—C9	117.48 (15)	C5—C6—H6	120.4
N1—C1—C2^i^	121.60 (14)	C8—C7—C6	119.26 (16)
N1—C1—C3	115.29 (14)	C8—C7—H7	120.4
C2^i^—C1—C3	123.11 (14)	C6—C7—H7	120.4
N1—C2—C1^i^	118.73 (14)	C7—C8—C9	118.03 (16)
N1—C2—C10	115.57 (14)	C7—C8—H8	121.0
C1^i^—C2—C10	125.69 (15)	C9—C8—H8	121.0
O1—C3—N2	122.72 (16)	N3—C9—C8	123.91 (17)
O1—C3—C1	122.48 (15)	N3—C9—H9	118.0
N2—C3—C1	114.80 (14)	C8—C9—H9	118.0
N2—C4—C5	115.02 (14)	C2—C10—H10A	109.5
N2—C4—H4A	108.5	C2—C10—H10B	109.5
C5—C4—H4A	108.5	H10A—C10—H10B	109.5
N2—C4—H4B	108.5	C2—C10—H10C	109.5
C5—C4—H4B	108.5	H10A—C10—H10C	109.5
H4A—C4—H4B	107.5	H10B—C10—H10C	109.5
N3—C5—C6	122.01 (15)		
			
C2—N1—C1—C2^i^	0.3 (3)	C9—N3—C5—C6	2.7 (2)
C2—N1—C1—C3	179.38 (14)	C9—N3—C5—C4	−176.51 (16)
C1—N1—C2—C1^i^	−0.3 (2)	N2—C4—C5—N3	−177.14 (14)
C1—N1—C2—C10	179.58 (15)	N2—C4—C5—C6	3.6 (2)
C4—N2—C3—O1	3.9 (3)	N3—C5—C6—C7	−2.3 (3)
C4—N2—C3—C1	−176.38 (14)	C4—C5—C6—C7	176.86 (15)
N1—C1—C3—O1	−178.22 (15)	C5—C6—C7—C8	0.1 (3)
C2^i^—C1—C3—O1	0.9 (3)	C6—C7—C8—C9	1.4 (3)
N1—C1—C3—N2	2.0 (2)	C5—N3—C9—C8	−1.1 (3)
C2^i^—C1—C3—N2	−178.88 (15)	C7—C8—C9—N3	−1.0 (3)
C3—N2—C4—C5	−83.2 (2)		

Symmetry code: (i) −*x*+2, −*y*+2, −*z*+2.

### (II) 3,6-Dimethyl-N2,N5-bis(pyridin-2-ylmethyl)pyrazine-2,5-dicarboxamide Hydrogen-bond geometry (Å, º)

**Table:**

*D*—H···*A*	*D*—H	H···*A*	*D*···*A*	*D*—H···*A*
N2—H2*N*···N3^ii^	0.85 (2)	2.35 (2)	3.097 (2)	147.4 (18)
C7—H7···O1^iii^	0.93	2.59	3.263 (2)	130

Symmetry codes: (ii) −*x*+3/2, *y*+1/2, −*z*+3/2; (iii) −*x*+5/2, *y*+1/2, −*z*+3/2.

### (III) N2,N5-Bis(pyridin-4-ylmethyl)pyrazine-2,5-dicarboxamide Crystal data

**Table:**

C_18_H_16_N_6_O_2_	*F*(000) = 364
*M**_r_* = 348.37	*D*_x_ = 1.472 Mg m^−^^3^
Monoclinic, *P*2_1_/*c*	Mo *K*α radiation, λ = 0.71073 Å
*a* = 5.8663 (6) Å	Cell parameters from 5301 reflections
*b* = 18.7539 (17) Å	θ = 2.2–25.9°
*c* = 7.2943 (8) Å	µ = 0.10 mm^−^^1^
β = 101.606 (12)°	*T* = 153 K
*V* = 786.08 (14) Å^3^	Block, colourless
*Z* = 2	0.35 × 0.30 × 0.25 mm

### (III) N2,N5-Bis(pyridin-4-ylmethyl)pyrazine-2,5-dicarboxamide Data collection

**Table:**

Stoe IPDS 1 diffractometer	1513 independent reflections
Radiation source: fine-focus sealed tube	1259 reflections with *I* > 2σ(*I*)
Plane graphite monochromator	*R*_int_ = 0.026
φ rotation scans	θ_max_ = 25.9°, θ_min_ = 2.2°
Absorption correction: multi-scan (MULABS; Spek, 2009)	*h* = −7→7
*T*_min_ = 0.962, *T*_max_ = 1.000	*k* = −22→23
5980 measured reflections	*l* = −8→8

### (III) N2,N5-Bis(pyridin-4-ylmethyl)pyrazine-2,5-dicarboxamide Refinement

**Table:**

Refinement on *F*^2^	Primary atom site location: structure-invariant direct methods
Least-squares matrix: full	Secondary atom site location: difference Fourier map
*R*[*F*^2^ > 2σ(*F*^2^)] = 0.036	Hydrogen site location: mixed
*wR*(*F*^2^) = 0.097	H atoms treated by a mixture of independent and constrained refinement
*S* = 1.08	*w* = 1/[σ^2^(*F*_o_^2^) + (0.0502*P*)^2^ + 0.2467*P*] where *P* = (*F*_o_^2^ + 2*F*_c_^2^)/3
1513 reflections	(Δ/σ)_max_ < 0.001
122 parameters	Δρ_max_ = 0.44 e Å^−^^3^
0 restraints	Δρ_min_ = −0.19 e Å^−^^3^

### (III) N2,N5-Bis(pyridin-4-ylmethyl)pyrazine-2,5-dicarboxamide Special details

**Table:**

Geometry. All esds (except the esd in the dihedral angle between two l.s. planes) are estimated using the full covariance matrix. The cell esds are taken into account individually in the estimation of esds in distances, angles and torsion angles; correlations between esds in cell parameters are only used when they are defined by crystal symmetry. An approximate (isotropic) treatment of cell esds is used for estimating esds involving l.s. planes.

### (III) N2,N5-Bis(pyridin-4-ylmethyl)pyrazine-2,5-dicarboxamide Fractional atomic coordinates and isotropic or equivalent isotropic displacement parameters (Å^2^)

**Table:**

	*x*	*y*	*z*	*U*_iso_*/*U*_eq_	
N1	0.30168 (19)	0.46198 (6)	1.02233 (16)	0.0173 (3)	
N2	−0.00374 (19)	0.41317 (6)	0.72012 (17)	0.0187 (3)	
H2N	−0.029 (3)	0.4166 (10)	0.841 (3)	0.039 (5)*	
N3	−0.2298 (2)	0.15345 (7)	0.55300 (19)	0.0293 (3)	
O1	0.24963 (17)	0.44020 (6)	0.53216 (14)	0.0265 (3)	
C1	0.3536 (2)	0.47275 (7)	0.85410 (18)	0.0160 (3)	
C2	0.4500 (2)	0.48929 (7)	1.16888 (19)	0.0178 (3)	
H2	0.420845	0.482665	1.291218	0.021*	
C3	0.1942 (2)	0.44098 (7)	0.68574 (19)	0.0172 (3)	
C4	−0.1807 (2)	0.38348 (7)	0.5713 (2)	0.0203 (3)	
H4A	−0.148718	0.399204	0.449379	0.024*	
H4B	−0.334049	0.403073	0.582431	0.024*	
C5	−0.1943 (2)	0.30298 (7)	0.57217 (19)	0.0188 (3)	
C6	−0.4033 (3)	0.26981 (8)	0.4972 (2)	0.0243 (3)	
H6	−0.538854	0.297195	0.450754	0.029*	
C7	−0.4112 (3)	0.19603 (8)	0.4913 (2)	0.0300 (4)	
H7	−0.555967	0.174192	0.439677	0.036*	
C8	−0.0300 (3)	0.18638 (8)	0.6274 (2)	0.0263 (3)	
H8	0.102255	0.157690	0.674601	0.032*	
C9	−0.0045 (2)	0.25969 (8)	0.6396 (2)	0.0233 (3)	
H9	0.141680	0.280149	0.693639	0.028*	

### (III) N2,N5-Bis(pyridin-4-ylmethyl)pyrazine-2,5-dicarboxamide Atomic displacement parameters (Å^2^)

**Table:**

	*U*^11^	*U*^22^	*U*^33^	*U*^12^	*U*^13^	*U*^23^
N1	0.0187 (6)	0.0146 (5)	0.0189 (6)	−0.0010 (4)	0.0041 (5)	−0.0002 (4)
N2	0.0183 (6)	0.0194 (6)	0.0182 (6)	−0.0038 (5)	0.0033 (5)	−0.0020 (5)
N3	0.0346 (7)	0.0212 (6)	0.0303 (7)	−0.0015 (5)	0.0023 (6)	−0.0005 (6)
O1	0.0264 (5)	0.0345 (6)	0.0193 (5)	−0.0081 (4)	0.0063 (4)	−0.0033 (4)
C1	0.0172 (6)	0.0121 (6)	0.0185 (7)	0.0017 (5)	0.0032 (5)	−0.0002 (5)
C2	0.0203 (7)	0.0159 (7)	0.0175 (7)	−0.0007 (5)	0.0044 (5)	0.0007 (5)
C3	0.0189 (6)	0.0131 (6)	0.0194 (7)	0.0004 (5)	0.0030 (5)	−0.0003 (5)
C4	0.0192 (7)	0.0184 (7)	0.0216 (7)	−0.0015 (5)	0.0001 (5)	−0.0012 (6)
C5	0.0206 (7)	0.0195 (7)	0.0168 (7)	−0.0013 (5)	0.0049 (5)	−0.0015 (5)
C6	0.0209 (7)	0.0217 (7)	0.0283 (8)	−0.0012 (6)	0.0005 (6)	−0.0006 (6)
C7	0.0292 (8)	0.0225 (8)	0.0358 (9)	−0.0061 (6)	0.0009 (7)	−0.0015 (6)
C8	0.0280 (8)	0.0242 (8)	0.0256 (8)	0.0049 (6)	0.0029 (6)	−0.0011 (6)
C9	0.0197 (7)	0.0246 (8)	0.0248 (7)	−0.0014 (6)	0.0022 (6)	−0.0029 (6)

### (III) N2,N5-Bis(pyridin-4-ylmethyl)pyrazine-2,5-dicarboxamide Geometric parameters (Å, º)

**Table:**

N1—C2	1.3372 (18)	C4—C5	1.5120 (19)
N1—C1	1.3378 (18)	C4—H4A	0.9900
N2—C3	1.3417 (18)	C4—H4B	0.9900
N2—C4	1.4536 (17)	C5—C9	1.385 (2)
N2—H2N	0.93 (2)	C5—C6	1.386 (2)
N3—C7	1.334 (2)	C6—C7	1.385 (2)
N3—C8	1.339 (2)	C6—H6	0.9500
O1—C3	1.2278 (17)	C7—H7	0.9500
C1—C2^i^	1.3928 (19)	C8—C9	1.384 (2)
C1—C3	1.5085 (18)	C8—H8	0.9500
C2—H2	0.9500	C9—H9	0.9500
			
C2—N1—C1	116.35 (12)	C5—C4—H4B	108.7
C3—N2—C4	121.62 (12)	H4A—C4—H4B	107.6
C3—N2—H2N	117.3 (12)	C9—C5—C6	117.45 (13)
C4—N2—H2N	120.9 (12)	C9—C5—C4	123.19 (12)
C7—N3—C8	115.74 (13)	C6—C5—C4	119.32 (12)
N1—C1—C2^i^	122.28 (12)	C7—C6—C5	118.82 (14)
N1—C1—C3	117.96 (12)	C7—C6—H6	120.6
C2^i^—C1—C3	119.75 (12)	C5—C6—H6	120.6
N1—C2—C1^i^	121.37 (13)	N3—C7—C6	124.63 (14)
N1—C2—H2	119.3	N3—C7—H7	117.7
C1^i^—C2—H2	119.3	C6—C7—H7	117.7
O1—C3—N2	124.33 (13)	N3—C8—C9	123.95 (14)
O1—C3—C1	120.85 (12)	N3—C8—H8	118.0
N2—C3—C1	114.80 (12)	C9—C8—H8	118.0
N2—C4—C5	114.18 (11)	C8—C9—C5	119.40 (13)
N2—C4—H4A	108.7	C8—C9—H9	120.3
C5—C4—H4A	108.7	C5—C9—H9	120.3
N2—C4—H4B	108.7		
			
C2—N1—C1—C2^i^	−0.5 (2)	N2—C4—C5—C9	−29.1 (2)
C2—N1—C1—C3	178.30 (11)	N2—C4—C5—C6	152.96 (13)
C1—N1—C2—C1^i^	0.5 (2)	C9—C5—C6—C7	−0.8 (2)
C4—N2—C3—O1	−4.5 (2)	C4—C5—C6—C7	177.20 (14)
C4—N2—C3—C1	177.11 (11)	C8—N3—C7—C6	1.0 (2)
N1—C1—C3—O1	−169.52 (12)	C5—C6—C7—N3	−0.1 (3)
C2^i^—C1—C3—O1	9.29 (19)	C7—N3—C8—C9	−1.0 (2)
N1—C1—C3—N2	8.95 (17)	N3—C8—C9—C5	0.1 (2)
C2^i^—C1—C3—N2	−172.24 (12)	C6—C5—C9—C8	0.8 (2)
C3—N2—C4—C5	106.64 (15)	C4—C5—C9—C8	−177.11 (14)

Symmetry code: (i) −*x*+1, −*y*+1, −*z*+2.

### (III) N2,N5-Bis(pyridin-4-ylmethyl)pyrazine-2,5-dicarboxamide Hydrogen-bond geometry (Å, º)

**Table:**

*D*—H···*A*	*D*—H	H···*A*	*D*···*A*	*D*—H···*A*
N2—H2*N*···N3^ii^	0.93 (2)	2.50 (2)	3.2420 (19)	137.5 (16)
C2—H2···O1^iii^	0.95	2.33	3.2411 (18)	160
C4—H4*B*···O1^iv^	0.99	2.49	3.4636 (18)	166

Symmetry codes: (ii) *x*, −*y*+1/2, *z*+1/2; (iii) *x*, *y*, *z*+1; (iv) *x*−1, *y*, *z*.
